# *OsWRKY53* Promotes Abscisic Acid Accumulation to Accelerate Leaf Senescence and Inhibit Seed Germination by Downregulating Abscisic Acid Catabolic Genes in Rice

**DOI:** 10.3389/fpls.2021.816156

**Published:** 2022-01-27

**Authors:** Wenya Xie, Xinru Li, Shiping Wang, Meng Yuan

**Affiliations:** National Key Laboratory of Crop Genetic Improvement, National Center of Plant Gene Research, Huazhong Agricultural University, Wuhan, China

**Keywords:** leaf senescence, seed germination, ABA, *OsWRKY53*, transcription factor

## Abstract

Abscisic acid (ABA) largely promotes leaf senescence and inhibits seed germination in plants. Endogenous ABA content is finely tuned by many transcription factors. In this study, we showed that *OsWRKY53* is a positive regulator of leaf senescence and a negative regulator of seed germination in rice. *OsWRKY53* expression was induced in leaves under aging, dark, and ABA treatment. The *OsWRKY53*-overexpressing (*OsWRKY53*-oe) plants showed early yellowing leaves, while the *OsWRKY53* (*oswrky53*) knockout mutants maintained green leaves than the wild type under natural, dark-induced, and ABA-induced senescence conditions. Transcriptional analysis revealed that ABA catabolic genes, namely, *OsABA8ox1* and *OsABA8ox2*, two key genes participating in ABA catabolism harboring ABA 8′-hydroxylase activity, were markedly downregulated in *OsWRKY53*-oe leaves. Chromatin immunoprecipitation and protoplast transient assays revealed that OsWRKY53 directly bound to the promoters of *OsABA8ox1* and *OsABA8ox2* to repress their transcription, resulting in elevated endogenous ABA contents that promoted premature leaf senescence in the *OsWRKY53*-oe plants. It indicates that OsWRKY53 is a positive regulator through regulating ABA accumulation to promote leaf senescence. In addition, accumulated ABA simultaneously inhibited seed germination and post-germination growth in *OsWRKY53*-oe plants. Taken together, OsWRKY53 suppresses the transcript of ABA catabolic genes to promote ABA accumulation to modulate ABA-induced leaf senescence and ABA-mediated inhibition of seed germination.

## Introduction

Leaf senescence, the final stage of leaf development, is featured with developmentally programmed leaves yellowing due to the loss of green pigment chlorophyll (Lim et al., 2007; Woo et al., 2019). The onset of leaf senescence begins with chlorophyll degradation, while accelerated chlorophyll degradation usually promotes leaf senescence. Leaf senescence is controlled by multiple genetic and environmental factors. The major genetic factors are termed senescence-associated genes (SAGs), which exhibit accumulated gene expressions during leaf senescence. So far, many SAGs have been characterized in rice. They are involved in nutrient relocation, macromolecules degradation, transcriptional regulation, and signal transduction (Lee and Masclaux-Daubresse, 2021). Rice plants containing these loss-of-function or gain-of-function SAGs usually exhibit delayed or accelerated leaf senescence. Moreover, environmental factors including nutrient status, light quality and length, climate change, water usage, and biotic stress separately or collaboratively modulate leaf senescence (Lee and Masclaux-Daubresse, 2021).

Leaf senescence can be triggered or regulated by a variety of phytohormones, with abscisic acid (ABA), ethylene, jasmonic acid (JA), salicylic acid, brassinosteroid, and strigolactone functioning as inducers, whereas cytokinin, gibberellic acid (GA), and auxin functioning as inhibitors (Lim et al., 2007; Woo et al., 2019). Of these phytohormones, ABA plays a key role in plant to promote leaf senescence. Since ABA concentration is controlled by ABA biosynthetic genes or ABA catabolic genes in plants (Nambara and Marion-Poll, 2005), disruption of ABA catabolic genes or overexpression of ABA biosynthetic genes usually promotes leaf senescence, whereas downregulation of ABA biosynthetic genes or activation of ABA catabolic genes frequently delays leaf senescence (Yang et al., 2014; Gao et al., 2016; Huang et al., 2019). Moreover, ABA can specifically induce the expression of senescence-associated transcription factors to promote leaf senescence, which is termed ABA-induced leaf senescence. ABA elevates the transcripts of three transcription factors, namely, *ABF2*, *ABF3*, and *ABF4*, to accelerate chlorophyll degradation, leading to leaf senescence in *Arabidopsis* (Gao et al., 2016), and induces the expression of transcription factor *OsNAP* to promote leaf senescence in rice (Liang et al., 2014). Apart from promoting leaf senescence, ABA can inhibit seed germination. Several rice ABA biosynthetic and catabolic genes have been reported to affect seed germination-related phenotype (Sano and Marion-Poll, 2021). For example, ABA biosynthetic gene *OsNCED3* involving in ABA accumulation and ABA catabolic gene *OsABA8ox1* participating in ABA degradation play essential roles in seed germination or post-germination growth (Mao et al., 2017; Huang et al., 2018; Yu et al., 2021).

Transcription factors including NAC, WRKY, and MYB proteins have been referenced regulating leaf senescence. Transcriptions of these transcription factors are induced or suppressed during the process of leaf senescence (Kim et al., 2016). They positively or negatively modulate leaf senescence by regulating downstream target genes. In rice, OsNAC2 directly suppresses *OsABA8ox1* transcript, leading to ABA accumulation that triggers ABA-induced leaf senescence (Mao et al., 2017). OsNAP activates the transcripts of chlorophyll degradation genes, such as *RCCR1*, *SGR*, *NYC3*, and *NYC1*, resulting in leaf senescence (Liang et al., 2014). Among the 98 OsWRKYs in rice, *OsWRKY5*, *OsWRKY23*, *OsWRKY42*, and *OsWRKY93* have been characterized as senescence-related genes, promoting or inhibiting leaf senescence (Jing et al., 2009; Han et al., 2014; Kim et al., 2019; Li et al., 2021). Other 33 *OsWRKYs* expressions are induced or repressed during leaf senescence (Li et al., 2021), whether they modulate leaf senescence is largely unknown.

*OsWRKY53* has been reported to play critical roles in rice growth and development by regulating plant architecture and seed size (Tian et al., 2017, 2021). It also plays roles in plant responses to biotic stress, including pathogens or herbivore, and abiotic stress, such as wounding (Chujo et al., 2007; Yoo et al., 2014; Hu et al., 2015; Xie et al., 2021). In this study, we found that *OsWRKY53* promotes premature leaf senescence and inhibits seed germination by modulating ABA metabolism. By integrating genetic assays and biochemical experiments, we uncovered the molecular mechanism of *OsWRKY53*-regulated leaf senescence and seed germination with that OsWRKY53 directly bound to the promoters of *OsABA8ox1* and *OsABA8ox2* to repress their expression, resulting in ABA accumulation, which in turn accelerates leaf senescence and inhibits seed germination.

## Materials and Methods

### Plant Materials and Treatments

Plant materials used in this study were *OsWRKY53*-overexpressing (*OsWRKY53*-oe) plants, *OsWRKY53* (*oswrky53*) mutants, *OsWRKY53*-GFP plants, and the corresponding wild-type cultivar Zhonghua 11 (ZH11) (Xie et al., 2021). The seeds were sown on seedbeds, and 1 month later, the seedlings were transplanted to the paddy field. For dark-induced senescence experiments, detached leaves from 2-month-old plants were cut into 1-cm leaf disks and incubated in complete darkness with 50-μM ABA treatment, or a mock treatment with water only.

### Seed Germination and Post-Germination Growth Assays

The seeds of *OsWRKY53*-oe, *oswrky53*, and wild type were harvested at 30 days after heading, threshed and soaked in distilled water, and then spread onto plates covered with wet filter papers. To detect ABA effect on seed germination, seeds of consistent maturity were spread onto plates containing 20 ml of distilled water or 4 μM ABA solution. The plates were placed in a chamber under a 14-h light/10-h dark cycle at 28°C. Germination was defined as the emergence of the radical, and the number of germinated seeds was counted every 24 h. Germination rate was calculated as the number of total germinated seeds divided by the number of total seeds spread. Post-germination growth was evaluated by measuring shoot length of seedlings.

### Chlorophyll Measurement

About 50 mg leaves from *OsWRKY53*-oe, *oswrky53*, and wild-type plants at the booting stage were sampled for chlorophyll measurement. Rice leaves were incubated in extraction solution (acetone:absolute ethanol:water, 4.5:4.5:1, v/v/v) for 12 h, and the extraction was measured spectrophotometrically at 645 and 663 nm using a Spark™^M^ Multimode Microplate Reader (Tecan). The total chlorophyll (mg/g) = (20.29*A*_645_ + 8.05*A*_663_) × *v*/*m* × 1,000, where *v* is the volume of extraction solution, and *m* is the mass of leaves.

### Measurement of Ion Leakage Rates

The detached leaves were cut into 1-cm piece and immersed in 8 ml of deionized water in a 10-ml test tube for 24 h at room temperature with continual shaking at a speed of 100 rpm. The initial conductivity (R1) was measured using a Conductivity Meter (DDSJ-308A, Shanghai Leici, China). After that, the test tubes were placed in boiling water for 20 min and cooled naturally to room temperature, and the conductivity (R2) was documented again. Ion leakage rate was calculated as the ratio of R1 to R2.

### Abscisic Acid Measurement

Three-month-old leaves of *OsWRKY53*-oe, *oswrky53*, and wild-type plants were harvested for endogenous ABA measurement. Briefly, three replicates of frozen leaf samples (∼100 mg for each replicate) were pulverized in liquid nitrogen and then homogenized in 80% methanol. Each sample was extracted twice with extraction solution (methanol:water:glacial acetic acid, 80:19:1, v/v/v) *via* incubation for 12 h at 4°C and centrifuged at 5,000*g* for 30 min. The supernatant was filtered using a nylon filter with 0.22-μm pore size (Jinteng, China), and the eluate was measured using an ABA ELISA kit (Shanghai Jianglai Biotechnology, China) according to the instructions of the manufacturer.

### Gene Expression Analysis

For gene expression analysis, total RNA from the leaves of *OsWRKY53*-oe, *oswrky53*, and wild-type plants was isolated using Trizol reagent (Invitrogen, United States). cDNAs were synthesized with Superscript II reverse transcriptase (Invitrogen, United States) according to the manufacturer’s protocol. Quantitative real-time PCR (RT-qPCR) was performed using LightCycler 480 SYBR Green I Master (Roche, Switzerland) in the ABI 7500 Real-Time PCR System (Applied Biosystems, United States) as described previously (Xie et al., 2021). Gene-specific primers were designed using primer analysis software Primer Express version 3.0 (Applied Biosystems, United States). The transcript levels of rice *actin* gene were used to normalize expression levels for genes (Supplementary Table 1). The transcript levels of examined genes were quantified by a relative quantitation method (2^–ΔΔ*CT*^). Each RT-qPCR assay was biologically repeated at least twice with similar results, with each repetition having three technical replicates. The differences were analyzed for statistical significance using two-tailed Student’s *t*-test.

### Chromatin Immunoprecipitation-qPCR

Chromatin immunoprecipitation assay was performed as described previously (Xie et al., 2021). Briefly, chromatin was extracted and fragmented *via* ultrasound to 200–400 bp. Antibodies anti-GFP and IgG as control were incubated with 40 μl protein A Dynabeads (Invitrogen, Norway) at 4°C for 4 h after washing the beads, then 100 μl fragmented chromatin suspension was added, followed by incubation at 4°C for 12 h. After extensive washing and de-crosslinking, the precipitated and input DNA samples were analyzed by qPCR using gene-specific primers (Supplementary Table 1).

### Transient Expression Assay in Protoplasts

The coding sequence of *OsWRKY53* was amplified with gene-specific primers and cloned into the pU1301-GFP vector as the effector, with the empty pU1301-GFP vector as the control (Xie et al., 2021). The *OsABA8ox1* and *OsABA8ox2* promoters (1,200 bp upstream of the translational initiation site) from ZH11 were amplified with gene-specific primers (Supplementary Table 1). The amplified PCR products were cloned into the pGreenII-0800 vector as the reporters (Xie et al., 2021). To measure the transcriptional regulation activity, the effector and reporter constructs were co-transformed into rice protoplasts derived from 14-day-old seedlings of rice cultivar ZH11 as described previously (Xie et al., 2021). Rice protoplasts were isolated by digesting rice sheath strips in digestion solution (i.e., 10 mM MES, pH 5.7, 1 mM CaCl_2_, 0.6 M mannitol, 0.03% β-mercaptoethanol, 0.1% BSA, 0.75% macerozume R10, and 0.3% cellulase RS) for 4 h. For transformation, 3 μg of each plasmid was added together and gently mixed with 100 μl of protoplasts and 110 μl of PEG-CaCl_2_ solution. The transfected protoplasts were cultured for 12 h at 25°C in the dark and collected by centrifugation at 100*g* for 8 min and immediately utilized in the luciferase assay. Luciferase activities were measured using a Dual Luciferase Reporter Assay System (Promega, United States) according to the instructions of the manufacturer. The relative reporter gene expression levels were expressed as the ratio of firefly luciferase (LUC) to the renilla luciferase (REN).

### Electrophoretic Mobility Shift Assay

The coding sequence of *OsWRKY53* was amplified with gene-specific primers (Supplementary Table 1) and cloned into the pCold-TF vector (Xie et al., 2021). The recombinant plasmid was introduced to *E. coli* BL21 (DE3) cells and then the cells were induced with 0.2 mM isopropylthio-β-galactoside for 12 h at 16°C and collected by centrifugation. Recombinant His-OsWRKY53 protein was purified using Ni Sepharose 6 fast Flow (GE-Healthcare, United States). A single-stranded DNA oligonucleotide containing the canonical W-box of *OsABA8ox1* promoter was synthesized and labeled with 5-carboxyfluorescein (FAM) at its 5′-end (Shanghai Sangon, China). To generate double-stranded oligos, an equal amount of the complementary signal-stranded oligos was mixed, heated to 95°C for 2 min, and annealed by gradually cooling down to room temperature. To perform EMSA assay, purified His-OsWRKY53 protein was incubated with FAM-labeled double-stranded oligos and increasing amounts of the unlabeled oligonucleotides (10-, 50-, and 100-fold of labeled probes) at room temperature for 20 min. The samples were finally subjected to electrophoresis on 6% native acrylamide gels in 0.5 × Tris-borate-EDTA buffer under a 4°C electrical field of 110 V for 2 h. Fluorescence signaling was visualized by using FUJIFILM (FLA-5100, Japan).

## Results

### *OsWRKY53* Promotes Natural Senescence

**FIGURE 1:**
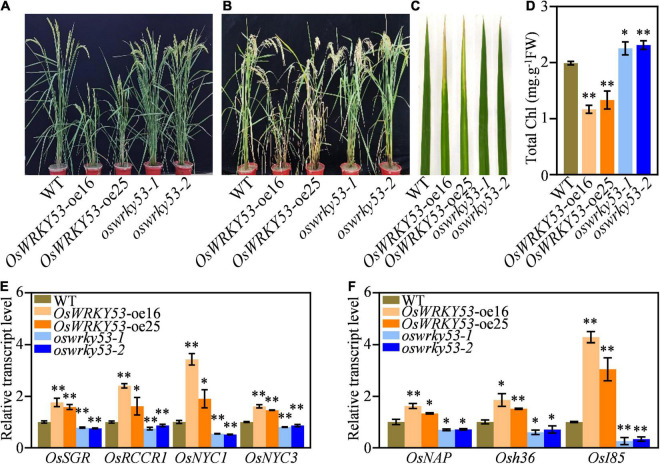
*OsWRKY53* promotes natural leaf senescence. **(A)** Phenotype of *OsWRKY53*-oe, *oswrky53*, and wild type (WT) at the heading stage. **(B)** Phenotype of *OsWRKY53*-oe, *oswrky53*, and WT at the grain-filling stage. **(C)** Senescing flag leaf of *OsWRKY53*-oe, *oswrky53*, and WT at the grain-filling stage. **(D)** Chlorophyll content of senescing flag leaf of *OsWRKY53*-oe, *oswrky53*, and WT at the grain-filling stage. **(E)** Transcript levels of chlorophyll degradation genes in rice leaves at the grain-filling stage. **(F)** Transcript levels of senescence-associated genes in rice leaves at the grain-filling stage. Data represent mean ± SD. Asterisks indicate a significant difference determined by two-tailed Student’s *t*-test at ^**^p < 0.01 or *p < 0.05.

We recently reported that *OsWRKY53* negatively regulates rice resistance to bacterial pathogen *via* thickening sclerenchyma cell walls (Xie et al., 2021). When we planted *OsWRKY53*-oe and *oswrky53* knockout plants in the paddy field, there were similar leaf development between *OsWRKY53*-oe, *oswrky53*, and wild type at the heading stage (Figure 1A), whereas *OsWRKY53*-oe plants showed accelerated leaf senescence and *oswrky53* mutants exhibited delayed leaf senescence compared with wild type at the grain-filling stage (Figure 1B). *OsWRKY53*-oe leaves were yellowish, while *oswrky53* and wild-type leaves retained green (Figure 1C). As leaf senescence is accompanied with reduced chlorophyll content, this promoted us to determine chlorophyll levels in leaves of these plants. Consistent with the visible accelerated leaf senescence, lower total chlorophyll contents were assessed in *OsWRKY53*-oe plants than that in *oswrky53* mutants and wild type (Figure 1D). We simultaneously assessed the transcript levels of representative chlorophyll degradation genes (e.g., *OsRCCR1*, *OsSGR*, *OsNYC3*, and *OsNYC1*) and SAGs (e.g., *OsNAP*, *Osh36*, and *OsI85*) in the leaves of these plants (Lee et al., 2001; Jiang et al., 2007; Kusaba et al., 2007; Morita et al., 2009; Tang et al., 2011; Liang et al., 2014; Shin et al., 2020). RT-qPCR assays showed that four chlorophyll degradation gene and three SAGs had higher transcript levels in *OsWRKY53*-oe plants, but lower levels in *oswrky53* mutants relative to them in wild type (Figures 1E,F). In line with OsWRKY53 regulating leaf senescence, *OsWRKY53* had the highest transcript levels in leaf and sheath rather than in other tissues (Supplementary Figure 1). Together, these results indicate that *OsWRKY53* is positively involved in natural leaf senescence.

### *OsWRKY53* Promotes Dark-Induced Senescence

**FIGURE 2:**
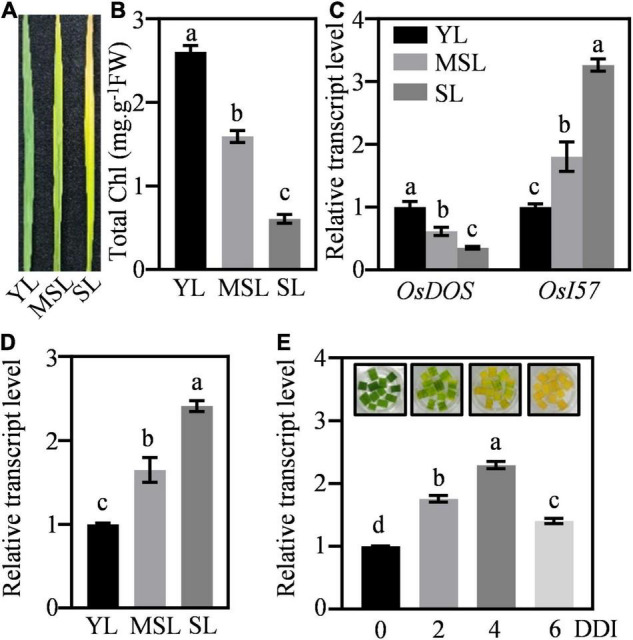
*OsWRKY53* promotes dark-induced senescence. **(A)** The appearance of rice leaves at different developmental stages. YL, young leaf. MSL, moderately senesced leaf. SL, senesced leaf. **(B)** Chlorophyll content in rice leaves at different developmental stages. **(C)** Transcript levels of senescence-related genes, namely, *OsDOS* and *OsI57*, in rice leaves at different developmental stages. **(D)** Transcript levels of *OsWRKY53* in rice leaves at different developmental stages. **(E)** Transcript levels of *OsWRKY53* in detached rice leaves during dark-induced senescence. DDI, day of dark incubation. The different letters above each bar indicate statistically significant differences determined by one-way ANOVA followed by Tukey’s multiple test (*p* < 0.05).

To further verify the role of *OsWRKY53* involving in leaf senescence, we assessed its transcriptional patterns in leaves under natural senescence. The transcript levels of *OsWRKY53* were detected in rice leaves at different developmental stages, from young leaves (YL), moderately senesced leaves (MSL), to senesced leaves (SL). The different expressions of marker SAGs (e.g., *OsDOS* and *OsI57*) and diverse chlorophyll contents in YL, MSL, and SL revealed the senescing status of these rice leaves (Figures 2A–C). The *OsWRKY53* transcripts were increased with leaf development, with the highest level in SL (Figure 2D). Then, we evaluated the response of *OsWRKY53* to dark-induced senescence. Along with darkness treatment, rice leaves were yellowish, exhibiting accelerated leaf senescence, and the transcript levels of *OsWRKY53* were significantly increased (Figure 2E), suggesting that OsWRKY53 may be a senescence-related transcription factor.

**FIGURE 3:**
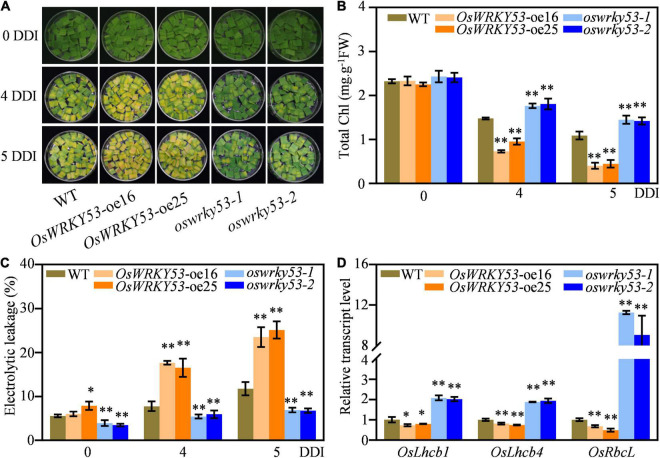
Overexpression of *OsWRKY53* promotes dark-induced senescence. **(A)** Phenotype of detached flag leaf of *OsWRKY53*-oe, *oswrky53*, and wild type (WT) at the booting stage after dark treatment. **(B)** Chlorophyll content of detached flag leaf of *OsWRKY53*-oe, *oswrky53*, and WT after dark treatment. **(C)** Ion leakage analysis of detached flag leaf of *OsWRKY53*-oe, *oswrky53*, and WT after dark treatment. **(D)** Relative transcript levels of photosystem-related genes or chlorophyll biosynthetic gene in detached flag leaf of *OsWRKY53*-oe, *oswrky53*, and WT after 5 DDI. DDI, day of dark incubation. Data represent mean ± SD. Asterisks indicate a significant difference between transgenic plants and WT determined by two-tailed Student’s *t*-test at ^**^p < 0.01 or *p < 0.05.

To explore accelerated leaf senescence caused by overexpression of *OsWRKY53* in more detail, we examined the responses of *OsWRKY53*-oe and *oswrky53* mutants under dark treatment. Detached leaf disks from *OsWRKY53*-oe, *oswrky53*, and wild-type plants at the booting stage were floated on MES solution and incubated in dark to mimic dark-induced senescence. After 4 days of dark incubation (DDI), the *OsWRKY53*-oe leaf disks showed yellowish, while *oswrky53* leaf disks retained green compared with light green of wild-type leaf disks. After 5 DDI, the accelerated yellowing tendency was greater in leaf disks of *OsWRKY53*-oe plants than that of wild type or *oswrky53* mutants (To explore accelerated leaf senescence caused by overexpression of *OsWRKY53* in more detail, we examined the responses of *OsWRKY53*-oe and *oswrky53* mutants under dark treatment. Detached leaf disks from *OsWRKY53*-oe, *oswrky53*, and wild-type plants at the booting stage were floated on MES solution and incubated in dark to mimic dark-induced senescence. After 4 days of dark incubation (DDI), the *OsWRKY53*-oe leaf disks showed yellowish, while *oswrky53* leaf disks retained green compared with light green of wild-type leaf disks. After 5 DDI, the accelerated yellowing tendency was greater in leaf disks of *OsWRKY53*-oe plants than that of wild type or *oswrky53* mutants (Figure 3A). Consistent with yellowing leaves, lower chlorophyll contents were observed for *OsWRKY53*-oe plants, and higher chlorophyll levels were determined for *oswrky53* mutants relative to that for wild type (Figure 3B). Accordingly, ion leakage rate, an indicator of membrane disintegration, was higher for *OsWRKY53*-oe plants, but lower for *oswrky53* mutants than wild type under darkness treatment for 4 or 5 days (Figure 3C). We also examined the transcript levels of photosystem-related genes, i.e., *OsLhcb1* and *OsLhcb4*, and chlorophyll biosynthetic gene, i.e., *OsRbcL*, in *OsWRKY53*-oe, *oswrky53*, and wild-type plants. The transcript levels of these genes were significantly lower in *OsWRKY53*-oe plants but higher in *oswrky53* mutants than in wild type after 5 DDI (Figure 3D). In parallel, 20-day seedlings of *OsWRKY53*-oe, *oswrky53*, and wild type were treated under darkness, with the similar results that *OsWRKY53*-oe plants exhibited accelerated leaf senescence (Supplementary Figure 2). Taken together, these results indicate that *OsWRKY53* promotes dark-induced senescence.

### Accumulated Abscisic Acid Contributes to Leaf Senescence for *OsWRKY53*-oe Plants

**FIGURE 4:**
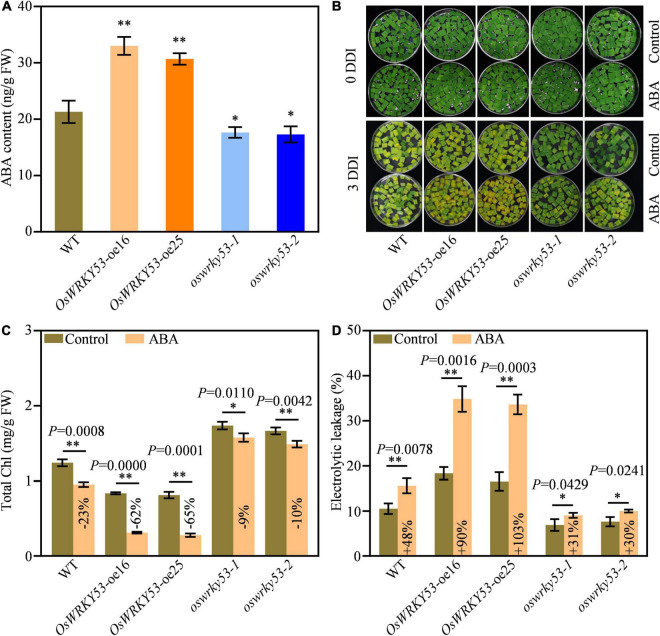
Accumulated ABA promotes leaf senescence in *OsWRKY53*-oe plants. **(A)** ABA contents in flag leaf of *OsWRKY53*-oe, *oswrky53*, and wild type (WT). Asterisks indicate a significant difference between transgenic plants and WT determined by two-tailed Student’s *t*-test at ^**^p < 0.01 or *p < 0.05. **(B)** Phenotype of detached flag leaf of *OsWRKY53*-oe, *oswrky53*, and WT at 3 days of dark incubation (DDI) in the presence of 50 mM ABA and control. **(C)** Chlorophyll content of detached flag leaf of *OsWRKY53*-oe, *oswrky53*, and WT at 3 DDI in the presence of 50 μM ABA and control. **(D)** Ion leakage analysis of detached flag leaf of *OsWRKY53*-oe, *oswrky53*, and WT at 3 DDI in the presence of 50 μM ABA and control. Data represent mean ± SD. Asterisks indicate a significant difference between exogenous ABA treatment and control determined by two-tailed Student’s *t*-test at ^**^p < 0.01 or *p < 0.05.

Leaf senescence is regulated by a variety of phytohormones, which act as senescence-promoting phytohormones or senescence-inhibiting phytohormones (Kusaba et al., 2013). To explore the mechanism of *OsWRKY53*-promoted leaf senescence, we analyzed expressions of phytohormones signaling or biosynthetic genes from the microarray data set of *OsWRKY53*-oe plants^1^, which has been deposited on public Gene Expression Omnibus (Chujo et al., 2014). A number of phytohormone signaling and biosynthetic genes have increased or decreased the transcript levels in *OsWRKY53*-oe plants relative to wild type (Supplementary Figure 3). Of these, majority of ABA signaling and biosynthetic genes have greater expressions in *OsWRKY53*-oe plants than in wild type, suggesting potential ABA accumulation in *OsWRKY53*-oe plants. We then measured ABA contents in the leaves of *OsWRKY53*-oe, *oswrky53*, and wild-type plants at the grain-filling stage. *OsWRKY53*-oe plants had 1.5-fold higher ABA level and *oswrky53* mutants had 1.3-fold lower ABA content than wild type (Figure 4A).

To explore whether *OsWRKY53* is involved in ABA-promoted leaf senescence, we assessed dynamic expression pattern of *OsWRKY53* under ABA treatment. Compared with mock control, the transcript levels of *OsWRKY53* were increased after exogenous ABA treatment, suggesting that OsWRKY53 is an ABA-activation transcription factor (Supplementary Figure 4). We also investigated leaf senescence of *OsWRKY53*-oe, *oswrky53*, and wild-type plants under exogenous ABA treatment. We found that exogenous ABA application accelerated leaf senescence for all the leaf disks after darkness treatment, whereas the accelerated senescing tendency was greater for *OsWRKY53*-oe plants than for *oswrky53* mutants and wild type at 3 DDI (Figure 4B). As a result, chlorophyll contents were decreased in all these plants; however, the decreased tendency was significantly greater for *OsWRKY53*-oe plants (−62% to 65%) than for *oswrky53* mutants (−9% to 10%) and wild type (−23%) after exogenous ABA treatment at 3 DDI (Figure 4C). Similarly, ion leakage rates were increased in all these plants, but the increased tendency was greater for *OsWRKY53*-oe plants (+ 90% to 103%) than for *oswrky53* mutants (+ 30% to 31%) and wild type (+ 48%) after exogenous ABA treatment at 3 DDI (Figure 4D). ABA treatment accelerated leaf senescence for *OsWRKY53*-oe plants compared with wild type and *oswrky53* mutants, indicating that overexpression of *OsWRKY53* enhances ABA-promoted leaf senescence. Taken together, these results indicate that *OsWRKY53* positively modulates ABA-induced leaf senescence.

### *OsWRKY53* Regulates Transcripts of Abscisic Acid Catabolic Genes

**FIGURE 5:**
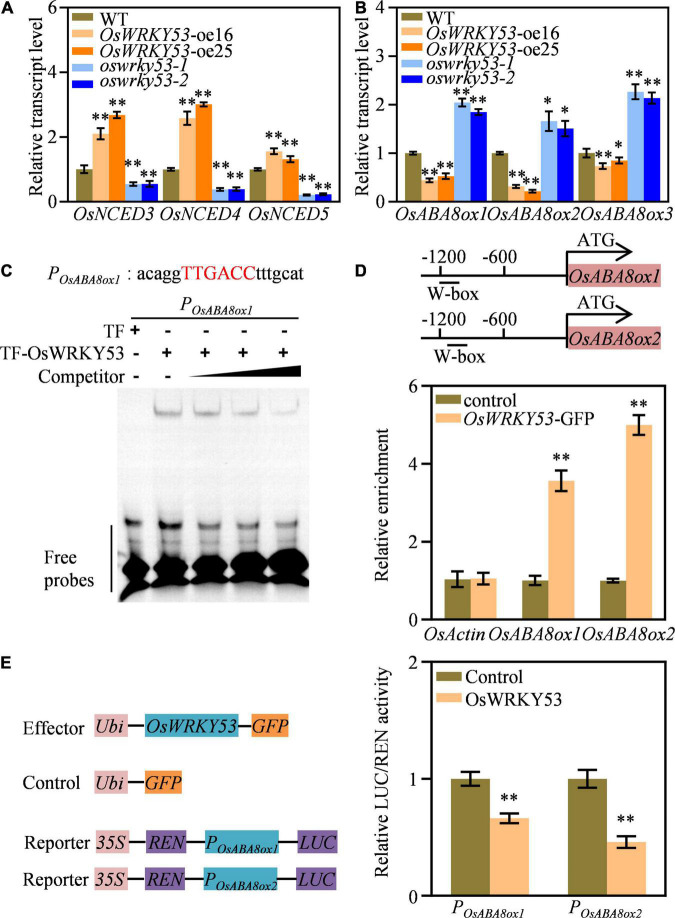
*OsWRKY53* binds and downregulates ABA catabolic genes expression. **(A)** Relative transcript levels of three ABA biosynthetic genes in *OsWRKY53*-oe, *oswrky53* mutants, and wild type (WT). **(B)** Relative transcript levels of three ABA catabolic genes in *OsWRKY53*-oe, *oswrky53* mutants, and WT. **(C)** DNA-binding activity assay of OsWRKY53 by EMSA. The red capital letters indicate the canonical W-box. **(D)** Binding assay of OsWRKY53 to the promoters of *OsABA8ox1* and *OsABA8ox2* by ChIP-qPCR in *OsWRKY53*-GFP plants using the anti-GFP antibody. **(E)** Activity assay of OsWRKY53 in regulating *OsABA8ox1* and *OsABA8ox2* expression. Data represent mean ± SD. Asterisks indicate a significant difference between transgenic lines and WT determined by two-tailed Student’s *t*-test at ^**^p < 0.01 or *p < 0.05 (**A–C**), or a significant difference between control and effector determined by two-tailed Student’s *t*-test ^**^P < 0.01 **(D)**.

The ABA accumulation in *OsWRKY53*-oe plants promoted us to investigate the transcript levels of ABA metabolism genes. We detected expressions of ABA biosynthetic genes (i.e., *OsNCED3*, *OsNCED4*, and *OsNCED5*) and ABA catabolic genes (i.e., *OsABA8ox1*, *OsABA8ox2*, and *OsABA8ox3*) in the leaves of *OsWRKY53*-oe, *oswrky53*, and wild-type plants. These three ABA biosynthetic genes had higher transcript levels in *OsWRKY53*-oe plants, but lower transcript levels in *oswrky53* mutants than in wild type (Figure 5A). Contrarily, three ABA catabolic genes had lower transcript levels in *OsWRKY53*-oe plants, but higher transcript levels in *oswrky53* mutants than in wild type (Figure 5B). The enhanced transcript of ABA biosynthetic genes and attenuated expression of ABA catabolic genes supported increased ABA contents in *OsWRKY53*-oe plants.

OsWRKY53 acts as a transcription repressor by binding to the canonical W-box with TTGACC core sequence at the promoter of its target genes (Xie et al., 2021) OsWRKY53 acts as a transcription repressor by binding to the canonical W-box with TTGACC core sequence at the promoter of its target genes (Xie et al., 2021). Thus, we analyzed whether the above three ABA catabolic genes have W-box at their promoters, and that both *OsABA8ox1* and *OsABA8ox2* have the canonical W-box at their promoters (Supplementary Figure 5). We carried out EMSA and ChIP assays to evaluate whether OsWRKY53 could directly bind to *OsABA8ox1* and *OsABA8ox2* promoters. The *in vitro* EMSA assays showed that purified TF-OsWRKY53 could bind to probe containing “TTGACC” element (Figure 5C). The *in vivo* ChIP-qPCR assays revealed that enrichment of OsWRKY53 was detected on the W-box-containing region at the promoters of *OsABA8ox1* and *OsABA8ox2* using anti-GFP antibody in *OsWRKY53*-GFP plants (Xie et al., 2021), but not on other regions without W-box, suggesting direct binding of OsWRKY53 to the promoters of *OsABA8ox1* and *OsABA8ox2* (Figure 5D). We also conducted transient expression assays to analyze OsWRKY53 action on the expressions of *OsABA8ox1* and *OsABA8ox2*. The LUC reporters driven by ∼1.2 kb promoter of *OsABA8ox1* (*ProOsABA8ox1:LUC*) and *OsABA8ox2* (*ProOsABA8ox2:LUC*) were co-transformed into rice protoplasts with effector constructs *ProUbi:GFP* and *ProUbi:OsWRKY53-GFP*. The LUC activities of *ProOsABA8ox1:LUC* and *ProOsABA8ox2:LUC* were repressed by *OsWRKY53-GFP*, but not by *ProUbi:GFP*, indicating suppressed transcripts of *OsABA8ox1* and *OsABA8ox2* by OsWRKY53 (Figure 5E). Thus, these results collectively indicate that OsWRKY53 directly binds to *OsABA8ox1* and *OsABA8ox2* promoters to repress their transcripts.

### Overexpression of *OsWRKY53* Inhibits Seed Germination and Post-Germination Growth

**FIGURE 6:**
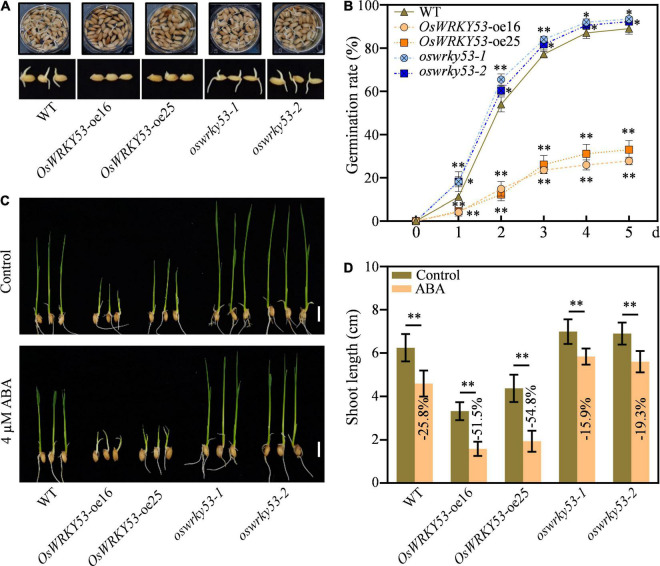
Overexpression of *OsWRKY53* inhibits seed germination and post-germination growth. **(A)** Phenotype of *OsWRKY53*-oe, *oswrky53* mutants, and wild type (WT) at 3 days after imbibition (DAI). **(B)** Seed germination rate of *OsWRKY53*-oe, *oswrky53* mutants, and WT under normal condition. The germination rate was counted every 24 h after imbibition. **(C)** Phenotype of seedlings of *OsWRKY53*-oe, *oswrky53* mutants, and WT at 10 DAI under 4 μM ABA treatment with water as a control. Scale bar = 1 cm. **(D)** Shoot length of *OsWRKY53*-oe, *oswrky53* mutants, and WT at 10 DAI under 4 μM ABA treatment with water as a control. Data represent mean ± SD. Asterisks indicate a significant difference between transgenic lines and WT determined by two-tailed Student’s *t*-test at ^**^p < 0.01 or *p < 0.05.

ABA not only promotes leaf senescence but also inhibits seed germination and post-germination growth (Gao et al., 2016; Yan and Chen, 2017; Song et al., 2020). To investigate the effect of ABA on germination performance for *OsWRKY53* transgenic lines, germination rates were examined using husked full seeds freshly harvested at 30 days after fertilization. Under normal conditions, the *oswrky53* mutants and wild-type seeds rapidly started to germinate after imbibition and the germination rate reached about 18.2 and 11.2%, respectively, at 1 day after imbibition (DAI), while the *OsWRKY53*-oe plants had only about 4.8% germination rate at 1 DAI (Figure 6A). At 3 DAI, the *oswrky53* mutants and wild-type seeds had germination rate over 82.5 and 77.1%, respectively, which were about threefold higher than that in *OsWRKY53*-oe plants. At 5 DAI, the final germination rate of *oswrky53* mutants and wild type were 92.9 and 89.1%, respectively, whereas the germination rate was 30.4% for *OsWRKY53*-oe plants (Figure 6B), suggesting that overexpression of *OsWRKY53* delays germination and knockout of *OsWRKY53* promotes germination.

To investigate the effect of ABA on post-germination growth for *OsWRKY53* transgenic lines, the shoot lengths were examined after seedlings of *OsWRKY53*-oe, *oswrky53*, and wild type were subjected to ABA treatment and non-ABA treatment for 10 days. Under non-ABA treatment, the shoot lengths for *OsWRKY53*-oe plants, *oswrky53* mutants, and wild type were 3.8, 7.0, and 6.2 cm, respectively (Figure 6C). The inhibited seedling growth for *OsWRKY53*-oe lines was consistent with accumulated ABA contents, and the improved seedling growth for *oswrky53* mutants was in line with attenuated ABA levels. Under ABA treatment, the shoot growths were inhibited for all these seedlings, and the shoot lengths for *OsWRKY53*-oe lines, *oswrky53* mutants, and wild type were 1.8, 5.7, and 4.6 cm, respectively (Figure 6C). The shoot growths were significantly hindered for *OsWRKY53*-oe plants (−51.5% to 54.8%), but slightly inhibited for *oswrky53* mutants (−15.9% to 19.3%) compared with wild type (−25.8%) (Figure 6D). ABA treatment significantly inhibited shoot growth for *OsWRKY53*-oe plants compared with wild type and *oswrky53* mutants, indicating that overexpression of *OsWRKY53* increases rice sensitivity to ABA. Collectively, these results indicate that accumulated ABA inhibits seed germination and post-germination growth in *OsWRKY53*-oe plants.

## Discussion

*OsWRKY53* has been previously reported to play critical roles not only in rice growth and development but also in responses to biotic and abiotic stress (Chujo et al., 2007; Yoo et al., 2014; Hu et al., 2015; Tian et al., 2017, 2021; Xie et al., 2021). In this study, we found that *OsWRKY53* has additional roles in leaf senescence and seed germination.

We found that *OsWRKY53* participates in ABA-induced leaf senescence. The transcript of *OsWRKY53* was activated by aging, dark, and ABA treatment (Figures 2D,E). In addition, seven W-boxes and one G-box in the 2,000-bp upstream of transcription start codon of *OsWRKY53* were identified (Supplementary Figure 6). These *cis*-elements are targeted by WRKY, bZIP, bHLH, and NAC transcription factors, which are involved in the regulation of leaf senescence (Liu et al., 2016). It seems that expression of *OsWRKY53* could be regulated by senescence-induced transcription factors or OsWRKY53 is possibly a senescence-induced transcription factor. Moreover, the progression of leaf senescence was significantly faster in *OsWRKY53*-oe plants than in wild type under natural, dark-induced, and ABA-induced senescence conditions, and the *oswrky53* knockout mutants showed delayed leaf senescence. These further indicate that *OsWRKY53* plays positive roles in leaf senescence.

Senescence-induced transcription factors directly or indirectly regulate expression of SAGs. By analyzing the differentially expressed genes (DEGs) from the microarray data set of *OsWRKY53*-oe plants, 591 genes were downregulated (Xie et al., 2021). Some of these downregulated DEGs may be the target genes of OsWRKY53 since OsWRKY53 acts as a transcription repressor. We found some senescence-related transcription factors, but not representative SAGs, from these downregulated DEGs, suggesting that OsWRKY53 may directly regulate expression of senescence-related transcription factors to modulate leaf senescence. However, we could not exclude the possibility that OsWRKY53 may indirectly regulate SAGs to promote leaf senescence, because some representative SAGs such as *OsNAP*, *Osh36*, and *OsI85* had higher expressions in *OsWRKY53*-oe plants than in wild type (Figure 1F). To investigate whether OsWRKY53 could directly bind to the promoters of senescence-related transcription factors, further biochemical, physiological, and genetic experiments should be carried out.

Apart from SAGs, senescence-related transcription factors can regulate expression of phytohormone metabolic genes to alter phytohormone homeostasis, leading to leaf senescence (Lim et al., 2007; Woo et al., 2019). We found that OsWRKY53 directly bound to the promoters of two ABA catabolic genes, namely, *OsABA8ox1* and O*sABA8ox2*, by recognizing the canonical W-box with EMSA and ChIP-qPCR assays (Figure 5), and suppressed the transcripts of *OsABA8ox1* and O*sABA8ox2* with transient expression and RT-qPCR assays (Figure 5). *OsABA8ox1* is a key gene in ABA catabolism and has ABA 8′-hydroxylase activity in rice. The *OsABA8ox1* knockout mutants accumulate ABA, exhibiting premature leaf senescence (Mao et al., 2017). Accordingly, decreased transcripts of these two ABA catabolic genes contributed to ABA accumulation, leading to accelerated leaf senescence for *OsWRKY53*-oe plants, whereas enhanced expressions of them caused less ABA content, leading to delayed leaf senescence for *oswrky53* mutants. It is consistent with that ABA is generally considered a senescence initiator (Lim et al., 2007; Woo et al., 2019). In addition, there were increased expressions of ABA biosynthetic genes such as *OsNCED3*, *OsNCED4*, and *OsNCED5* in *OsWRKY53*-oe plants relative to them in wild type, which may also contribute to ABA accumulation; however, they are not directly regulated by OsWRKY53. We speculate that these ABA biosynthetic genes may be the targets of senescence-related transcription factors, which are interacting proteins or downstream regulating genes of OsWRKY53. Based on present data, we conclude that OsWRKY53 downregulated transcripts of ABA metabolic genes and *OsWRKY53* functions in the promotion of leaf senescence by slowing ABA degradation.

Besides ABA and JA can promote leaf senescence. Several JA biosynthetic genes have higher expressions in the leaves of *OsWRKY53*-oe plants than in wild type (Supplementary Figure 3). Therefore, we could not exclude the possibility that JA-promoted leaf senescence simultaneously contributes to the accelerated leaf senescence for *OsWRKY53*-oe plants. Reversely, cytokinin and GA are senescence-inhibiting phytohormones. A number of biosynthetic genes of cytokinin and GA have decreased expressions in *OsWRKY53*-oe plants than in wild type. Whether OsWRKY53 directly binds and suppresses the expression of these biosynthetic genes, even less contents of cytokinin and GA contribute to the accelerated leaf senescence for *OsWRKY53*-oe plants should be further elucidated.

Abscisic acid is a negative regulator inhibiting seed germination and post-germination growth. In this study, the *OsWRKY53*-oe plants accumulating more ABA showed delayed germination after imbibition, lower germination rate, and retarded post-germination growth, whereas the *oswrky53* mutants containing less ABA exhibited fast germination, higher germination rate, and greater post-germination growth than wild type (Figure 6). In addition, the *OsWRKY53*-oe plants were more sensitive to exogenous ABA treatment, indicating that OsWRKY53 is a negative regulator of rice seed germination and post-germination growth.

Integrating previous reports and present results, OsWRKY53 has multiple roles, which is due to either different functions of downstream target genes that it regulates or diverse upstream signals to which it responds. OsWRKY53 plays positive roles in BR signaling, resistance to fungal blast, and a piercing-sucking herbivore and negative roles in resistance to bacterial blight and a chewing herbivore. Thus, the strategy that precisely modulates *OsWRKY53* expression to maximize its function for rice genetic improvement is a to be resolved and amazing question.

## Data Availability Statement

The original contributions presented in the study are included in the article/Supplementary Material, further inquiries can be directed to the corresponding author.

## Author Contributions

WX and XL performed the experiments. WX analyzed the data and drafted the manuscript. SW and MY supervised the project. MY drafted and revised the manuscript. All authors contributed to the article and approved the submitted version.

## Conflict of Interest

The authors declare that the research was conducted in the absence of any commercial or financial relationships that could be construed as a potential conflict of interest.

## Publisher’s Note

All claims expressed in this article are solely those of the authors and do not necessarily represent those of their affiliated organizations, or those of the publisher, the editors and the reviewers. Any product that may be evaluated in this article, or claim that may be made by its manufacturer, is not guaranteed or endorsed by the publisher.

## Footnotes

1
https://www.ncbi.nlm.nih.gov/geo/query/acc.cgi?acc=GSE48500
