# Supplementary material for: OsWRKY53 Promotes Abscisic Acid Accumulation to Accelerate Leaf Senescence and Inhibit Seed Germination by Downregulating Abscisic Acid Catabolic Genes in Rice
Source: Front Plant Sci. 2022 Jan 27;12:816156. doi: 10.3389/fpls.2021.816156 (PMC8828546; doi:10.3389/fpls.2021.816156)
Supplement: Supplementary file 1 [file Presentation_1.pdf]

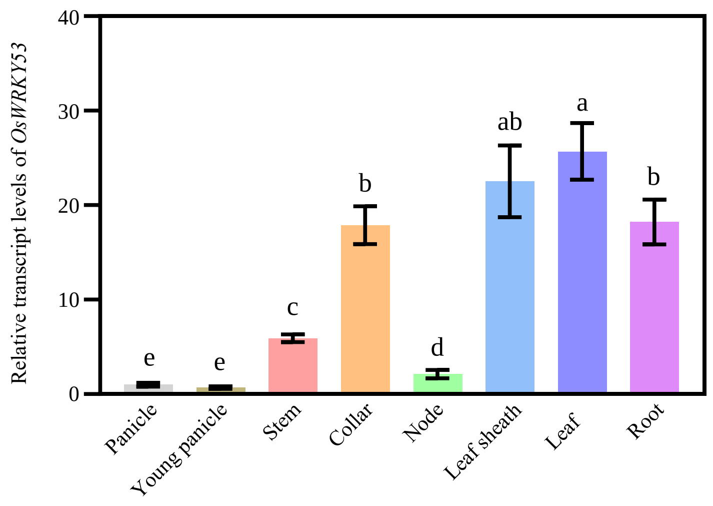

Supplementary Figure 1. Relative transcript levels for *OsWRKY53* in different tissues. Tissues were collected at the heading stage from cultivar ZH11. Date represent means ± SD. The different letters above each bar indicate statistically significant differences determined by one-way ANOVA analysis followed by Tukey's multiple test ($P < 0.05$).

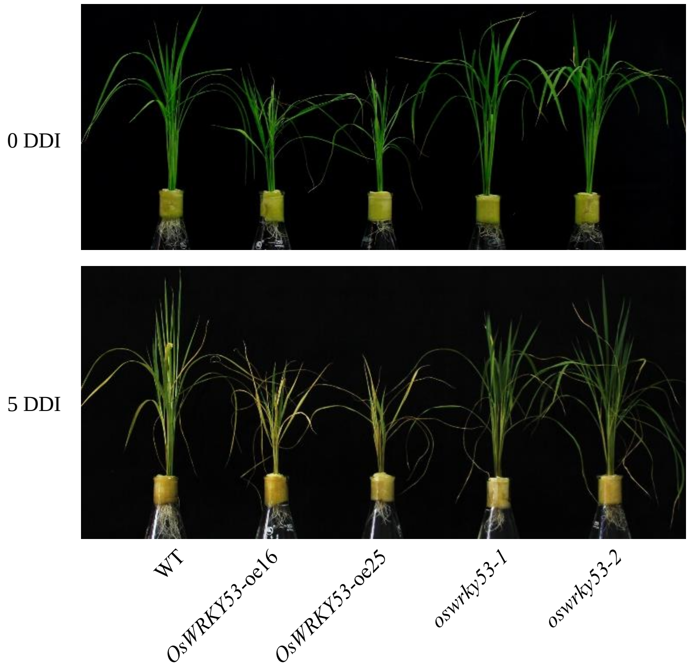

Supplementary Figure 2. Phenotype of 20-days seedlings of *OsWRKY53*-oe, *oswrky53*, and wild type (WT) after dark treatment. DDI, day of dark incubation.

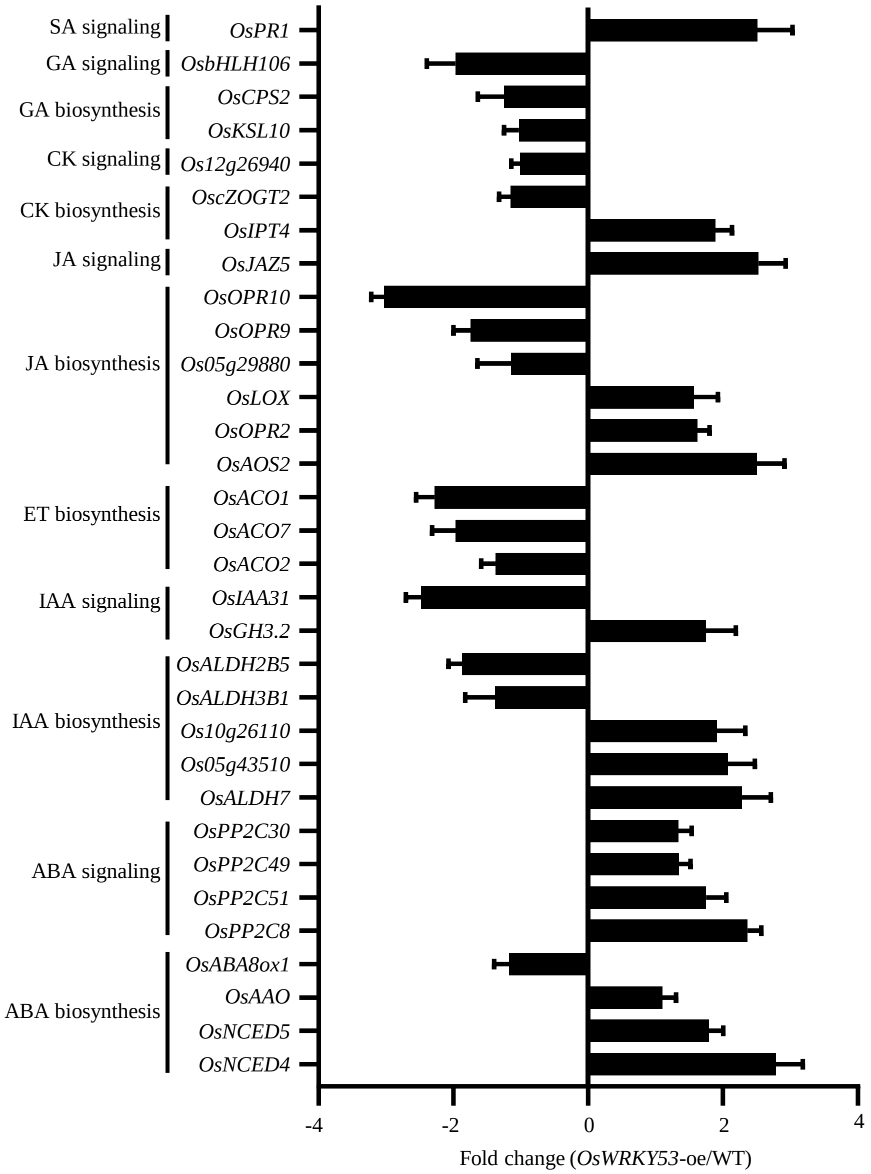

Supplementary Figure 3. The expression of signaling and biosynthetic genes for several phytohormones in *OsWRKY53*-overexpressing plants. The data were extracted and analyzed according to the public microarray database (https://www.ncbi.nlm.nih.gov/geo/query/acc.cgi?acc=GSE48500).

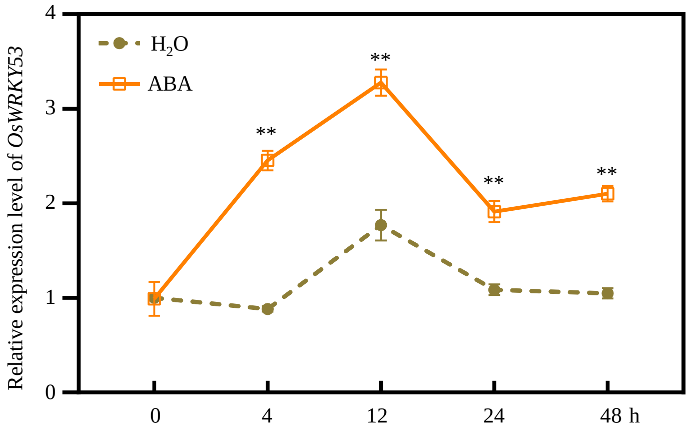

Supplementary Figure 4. The dynamic expression pattern of *OsWRKY53* after ABA treatment. Asterisks indicate a significant difference between H_2O and ABA treatment determined by two-tailed Student's t-test at $^{**}P < 0.01$.

P$_{OsABA8ox\text{-}1}$:

TTGATGAATAAGAGATGAATTGAAGCACACCCTCACAGCTGTCCCATCACATGCAAAGGTCAACC
TGTAGATCTGAATGCACATGCCTTTGAATTTCTGATTAAGATGTTAACCTACTACTAGAACTGCT
ACCATCCGATCTTTTTTTGCCCGAATCAAAAAAGAGAGAAAAGAGAAAAAAATATGCTTTTATTT
TTCTCAGAAGAAAAAAAGAGGGGTAGAGCTGCATGCGCACGACCATTTCGCGACGGACTAACGGT
TTGTTCAACACCTGTCATTCCCATCATGAGCAGGTTAAGCATTTCGGCACGCATTCCTACTTTGC
AACCTGCTTTAGCTAGCCGCGGTCAGTGATACCGGCAAAAATTCCCAAGTGATGTTTACTTCGAA
AAAATAGATTATTCACGCATCAATAAATCATAAAATGAGTATCTTACGATGGACTCAGGAACAAT
CGGTAGATGTGTTTACCGACAAAAAGAAAAAAAAAACACCGCCGCACCGGACACCAAGTAATTAA
GCAGAACAGTGTTCAGGCCAGTATGATGGCTGGTTGGGGATGATCAGAGGAAGCGGCCAAAGGAG
AGCAGCATTATAGGAGCAGGAGCAGGAGCAGGAGCAGCAGCAGCTGCTGCCGGGGCAGTACGTAG
TACGTACGAGAGCGCGTACAGTAGCTGATGGTGTGGAAACAGGGCGGAAAGGGAGAGGTGAGCTG
GAGAAAGGGATGATTCTCTCGCGCGCTGGCCAGAAGAGAAGAGAGGCCCATTTAGCTATCACTGT
ATTGGTTAACCGGTTTAACCGATCAGTTGATTAATCCGGCTCTCTCGCCCGCACGCCTTTTTCCC
TTTATTTCCGTATTGATTGCGCAATTATTCGGCAGCGTCTATCTGTCTCTCTCGCGCGCGGAGGC
ACCCAGCTCGGCTATTCTAGCGCGTAGTGGTGGTGCTAGCTACTAGTAGTAGTAGATTTTTGGGC
AGCAAGCGGCCGCAGAAAAGGAGAGCGAAACGGAGGAGAAACCGGGAAACTCAGCTCGCGGCCAA
TCTATAAATAGCTGCCACCCCTCTCGCCTTTCCCTCCACACCCCCCCAACAACACCACCGCCCA
TTTCCCTTTCTCTTCCTCTTCTTCTTCCTTCTCCTCCTACTCCTCTGCGATTGACAAAGATAAGT
GAAGTGAGCAGGCGCCAATGGGTGCTTTTCTTCTGTTCGTGTGCGTGCTCGCGCCTTTCTTGCTT
GTCTGC

P$_{OsABA8ox\text{-}2}$:

TTGTTGAATTAACTGAACAGTGGACACCTTTTTTTTCAAAAAAAATCTTTGACCAAATGAGAATA
GTTTAAAAGAAATGAAGAGAGCTGTGACAAAAGAGAGTACCGTCTTCATGCGGAAGAAATGGCAT
TAGAGGTTATACAAGTGGCAAGTAAAAATGGTCTAAACTTTATGGCAGGCTAGTAAAAAATAGTA
ATATAAACAACAACAGTCTAAGCAAAATTTAACTAAGGATTACAACAATCCATAATGATGTAGTA
CCAATCAAGCTGTCAGAGAGATCTCAGGCTCTGATGCTATGTTGAATCTCAATCCCCAAATCAAT
GCCTGTCCATGGGCCAAATCCCAGGGAATTCGTGCAAGCCCCGCTGAGCTGCACATGACTAGGAC
CCTATTTGAAAGATTAGCCCAGAACTAATTTTGAGAGCTAATATTTAGTAATGAATTGGTAGGCT
AAACATTAGTCTAGGTTGGTATGTTTGGATCTATGGGCTAATTCAAGGCTAAAAGGTAGAGAGAG
AGTAGAAAGAGAGGAGAGAGAAGGAGAGAGAGAGCTGCTTTTTGATGGTCCCCACACAAAATTAG
CCCCATTAGCACTTCTTAGAGAGGCTAATATTTTGAGAGGGGCTAATTCATATTAGCTCAAAATT
AGACAACCTGTTTGGATTCTTGAGAGCTAATTTAAGGCTAAAAGGTAAGAGCTAAACATTAGCCC
ATGGAAACAAACAGGGCCAGGAATCTTCTAATCTCCCTTTCATGTGTCCCTCCTTTTTTTCTCCC
TATCCCCAAAGATTTCACATTTTCTAATGAGCATCATGCCGTCCATGAGCTCTGTTTTTTATCCT
AACACCCCTCTGCAGCCGTGCTCACCCTCTATAAATACCCCATACAGGTGTCTTCCAATAGCACC
ACCATTTGGAAGAATCCTCCCCAAGATTGCTCGATCCCCTCGTGTCCCTCTCTCCCAACAACACT
GCGCCACAGACACAAATCACACTCAGATACGAAAGATAAGTACAGAGAGAGAGAGAGGGCACGGA
TTATACACTGCACACAAGCATATATATATATATATCGATTAGCCATCCGTGCTGATCTGAAGAGT
ATCATCGGTAGAGAGTTTTACAGAGTTGTTTGGACAAGGAGACACACACATGGCTTTCTTGCTCT
TCTTTGTCTTTGTGACAGCTGCAGTGCTGTGCTTCGTCGTCCCGGCGTTCTTGCTGCTCTGCACG
AGCGTG

Supplementary Figure 5. Distribution of the canonical W-box at the promoters of *OsABA8ox1* and *OsABA8ox2*. The W-box is indicated as red letters, and the transcription start codon is indicated as green letters.

GGAGTTTGGCACATTTGCATTCCGTCCATATTGCCTGATCAGTACGGCAGCGAAGATCGCC
GAGTTGCATGTCATTTTCCATAACCACATCGGCACATTGCACCACATCACACAAATTCATC
ACACATAACAAAAGAAAGACCACAACTTCAGTTCATAAAAAACTACTCTGTTCAAATGTTT
CGAGTTACCGCACACTGAAAAATTCCCGTGCAAAAATATCCTAAGTACAAGGTTTCCCGTG
CAATTTTACATAGATGAATGTGGAATTGAATTTGATGTGCAACTCTTATATTTTATTTCTA
TAAATATAAGTATGATTTCTATAAGAAACTGAAAATGGAATTGAATTTCTTGTACAACTCT
TGTGCATGCAATTGTAATTTGTATACAATATATTCTAAAAGGAGTATTGAATTCAGTATAA
AGCTCTTATCTTCTGTTTTGAGAGATATATATTGTACCTGGTATAGAACAATATCAGTCAA
TCTGTACCGTTAAAACCGTCAATATTTTTTTCTTCAATGTGAGAATTTCTAAGCCCATTTT
CGAGCTATCTTTTATAATATATTTCATCTGTTCTAAAATATAAGCATTTCCAAGATTCAAA
TATCAATATATGATGATCCAGTCTTTCAAAAGCGCTTATAGGGTAGGGTGTACGCGTGTTA
TGAGCGTCTATACTTGTTTTTCAACTAAATATATATGTAACAATATAAATATTCCTGCACT
AATTCCCATCATTTCAATGATTTAACATCCAATCAAATGCTAGAAAGGAAATGTAAGCAAT
TCAAAATTCAAAAATTGACAACTGAAGTAACAAAAATAATCTTTTGATCCAACCTTACTAC
TTAGGGGCTGTTTAATTCCCAAAACAAAAACTTTTCACCCATCACATCGAATGTTTGGACA
CATGCATGGAGTATTAATGTGAAAAAAAAATCAATTACACAGTTTGCATGTAAATTGCGAG
ACGAATCTTTTAAACCTAATTGCGCCAAGATTTACCAATATGGTGCTACAGTAAACATTTG
CTAATGACAGATTAATTAGACTTAACAAATTTGTTTTGCAGTTTCCTAGCGAAATCTGTAA
TTTGTTTTGTTATTAGACTACGTTTAATACAAATGTGTATCCGTATATCTAATGTAACTCG
CAAGGGCAAAAAAATTTTGCCAACTAAACAAACCCTTAAGAAGAACCTATAAATTGTTATA
CTTTAAAACAAAGAAAATAATAGATAGATATTATATGAGAGTGTGGTGCGCATAAGTAGAA
GTGTGGAAGATTGTTCTATATCTTCCGTGTAATGGAAAATACATCCAGTAATTATAGTTTT
TGATTTTAAAATTTCTCCTGATTTCTTCTCGAATAATTGCTCCGATACCTATCGCACAACA
ATTCCAAAGATGTGCTGTTGTTGAAGCACTGTATATATACAGCGTAATGAACGGGAAGTGT
TAACCATTTTATTAATACAAGGATATTTCATATGAAAAACCGAACAATAAAAAGGAAAAAG
CAAAAAAAAATCACATTTTTGTCTTTGTCCCCCCCAAATCCCCATCCCCAATCCGTCCGTC
CATTCGAAGCGACACGCCCACTAGTCCGCCCTCAACCCAAACCCAAACCCCAAGCTACACG
TACGAGTTGAAACTCGAAAGCCGTGGGAACACGCGACGCTCTCGGGTGGGCCCCACACCCC
GGCGTCTCCTTCCCCGAAACCACACGTCGTCGCCTCCCTCCCTCTCCTCTCACCTCCCGGC
GCGAGCCTCAGCCGCCTCGTCTGACCGGTCAAACCGCACGAGCTTTGACCTCACCCCCTTC
GCCGAAGCATCCCCAAACCATCACGTTCACATTAGCCTCCCTCTCCCACTGACACCCGGAC
CCCAACCTACTCCGCCCCACCTGTCAGAGACACGTGCCTCGCCTTTACGCTTCTGCCCCCA
CTGACCGGAGCAGCCGCTACTTAAGCCGCCGCGACACGAGCCTATCCACCACTCCCGTCTC
GTCGTCTCGTTCTCGTCTCCGATCACTCTCCTCCTCATCTTCGTCACGGTCTCCTCGCTTC
GCTAGCTCGCTTGCTTGCTGGCTGAGCTGTGGTACGCTCGCCATGGCGTCCTCGACGGGGG
GGTTGGACCACGGGTTCACGT

Supplementary Figure 6. Distributions of *cis*-elements at *OsWRKY53* promoter. The *cis*-elements were identified in the 2000-bp upstream of the transcription start codon. Red, yellow, and green shaded sequences represent the W-box, G-box, and start codon, respectively.

Supplementary Table 1. Primers used in this study.

Gene name	Forward primer (5'-3')	Reverse primer (5'-3')	Use
OsWRKY53	CCCGGCTCAGAGGTACGA	TGCTGCTCTTGCTCCTTGAA	RT-qPCR
OsNCED3	GTGGTGCTCGACAAGGAGAA	CAGAGGTGGAAGCAGAAGCA	RT-qPCR
OsNCED4	GAGGTACGACTTCCATGGGC	TTGAGGTACGGCTTGGACAC	RT-qPCR
OsNCED5	CCCAGCTTGAAGCTTTTGCT	ACAACACTGCAACTATCCCTATCACT	RT-qPCR
OsABA8ox1	AAGCTGGCAAAACCAACATC	CCGTGCTAATACGGAATCCA	RT-qPCR
OsABA8ox2	CTACTGCTGATGGTGGCTGA	CCCATGGCCTTTGCTTTAT	RT-qPCR
OsABA8ox3	AGTACAGCCCATTCCCTGTG	ACGCCTAATCAAACCATTGC	RT-qPCR
OsNAP	CAAGAAGCCGAACGGTTC	GTTAGAGTGGAGCAGCAT	RT-qPCR
OsNYC1	GAATCCGTAATTGGGCTGAA	CTGGAAGAGGTCCACCTGAG	RT-qPCR
OsNYC3	TGTCGTTGCCATGTGAAGAT	TTGGTCACGCCACAAATCTA	RT-qPCR
OsRCCR1	GGGATCGACGATTGATTTCATG	CACGCTGTTTGTCCACCTGAGT	RT-qPCR
OsSGR	AGGGGTGGTACAACAAGCTG	GCTCCTTGCGGAAGATGTAG	RT-qPCR
Osh36	GCACGGAGGCGAACGA	TTGAGCGGTAGCACCCATT	RT-qPCR
OsI85	GAGCAACGGCGTGGAGA	GCGGCGGTAGAGGAGATG	RT-qPCR
OsLhcb1	CCATGTTCTCCATGTTCGGCTTCT	TAGGCCCAGGCGTTGTTGTTGA	RT-qPCR
OsLcb4	TGCTCGTCATCGGCTACATC	CAGCCGCTCCTTCTTCTCC	RT-qPCR
OsRbcL	GGCAGCATTCCGAGTAAC	ATAGCATCGGCCTTTGTAA	RT-qPCR
OsABA8ox1	CTATAGGGCGAATTGATAAGAGATGAA TTGAAGCACACCC	CGCTCTAGAACTAGTCACTTCACTTATCTT TGTCAATCGC	LUC activity
OsABA8ox2	ACTATAGGGCGAATTGAACTGTTGTGC AAACTTCTTGATC	CGCTCTAGAACTAGTGTGTGTGTCTCCTT GTCCAAACAA	LUC activity
OsABA8ox1	CGGGATGAGTTGAACGACTGA	TCAAAGGCATGTGCATTCAGA	ChIP-qPCR
OsABA8ox2	CCGCATGAAGACGGTACTCT	ACTGAACAGTGGACACCTTTT	ChIP-qPCR
OsActin	TGTATGCCAGTGGTCGTACCA	CCAGCAAGGTCGAGACGAA	RT-qPCR
